# Indirect genetic effect model using feeding behaviour traits to define the degree of interaction between mates: an implementation in pigs growth rate

**DOI:** 10.1017/S1751731118001192

**Published:** 2018-06-06

**Authors:** M. Ragab, M. Piles, R. Quintanilla, J. P. Sánchez

**Affiliations:** 1 Genetica i Millora Animal, Institut de Recerca i Tecnologia Agroalimentàries (IRTA), Torre Marimon s/n, Caldes de Montbui, Barcelona, 08140, Spain; 2 Poultry Production Department, Kafr El-Sheikh University, Kafr El-Sheikh 33516, Egypt

**Keywords:** genetic parameters, social interactions, genetic selection, animal welfare, feeding behavior

## Abstract

An alternative implementation of the animal model including indirect genetic effect (IGE) is presented considering pair-mate-specific interaction degrees to improve the performance of the model. Data consisted of average daily gain (ADG) records from 663 pigs kept in groups of 10 to 14 mates during the fattening period. Three types of models were used to fit ADG data: (i) animal model (AM); (ii) AM with classical IGE (AM-IGE); and (iii) AM fitting IGE with a specific degree of interaction between each pair of mates (AM-IGE_i_). Several feeding behavior phenotypes were used to define the pair-mate-specific degree of interaction in AM-IGE_i_: feeding rate (g/min), feeding frequency (min/day), the time between consecutive visits to the feeder (min/day), occupation time (min/day) and an index considering all these variables. All models included systematic effects batch, initial age (covariate), final age (covariate), number of pigs per pen (covariate), plus the random effect of the pen. Estimated posterior mean (posterior SD) of heritability was 0.47 (0.15) using AM. Including social genetic effects in the model, total heritable variance expressed as a proportion of total phenotypic variance (*T* ^2^) was 0.54 (0.29) using AM-IGE, whereas it ranged from 0.51 to 0.55 (0.12 to 0.14) with AM-IGE_i_, depending on the behavior trait used to define social interactions. These results confirm the contribution of IGEs to the total heritable variation of ADG. Moreover, important differences between models were observed in EBV rankings. The percentage of coincidence of top 10% animals between AM and AM-IGE_i_ ranged from 0.44 to 0.89 and from 0.41to 0.68 between AM-IGE and AM-IGE_i_. Based on the goodness of fit and predictive ability, social models are preferred for the genetic evaluation of ADG. Among models including IGEs, when the pair-specific degree of interaction was defined using feeding behavior phenotypes we obtained an increase in the accuracy of genetic parameters estimates, the better goodness of fit and higher predictive ability. We conclude that feeding behavior variables can be used to measure the interaction between pen mates and to improve the performance of models including IGEs.

## Implications

When selecting traits that might be affected by the interaction between mates, important differences in ranking animals are observed between genetic evaluations with either standard models or models with indirect genetic effects (IGEs). Assuming that each individual interacts differently with its mates improves the performance of models fitting IGEs. This study shows how feeding behavior variables can be used to define a specific degree of interaction between each pair of animals, obtaining a more accurate genetic evaluation of traits affected by IGEs, such as growth.

## Introduction

Livestock animals are usually reared in groups. This housing system generates interaction between animals that is expected to increase if feed restriction is applied (Piles *et al*., 2017). In spite of that, interactions between individuals have been traditionally ignored in breeding programs (Wade, 1977; Craig and Muir, 1996; Bijma, 2010). In recent decades, interaction effects have received increased attention by both evolutionary biologists and animal breeders (Muir, 2005), as interaction effects could generate positive (cooperation) or negative (aggressive or competition) effects on animal welfare, productivity and health. A large body of literature reported that interactions among individuals can generate an additional level of heritable variation that affects direction and/or magnitude of selection response in traits affected by this type of IGEs (Wilson *et al*., 2009; Ellen *et al*., 2014). The main objective of animal models (AMs) including IGEs is separating direct genetic effects from IGEs which produced by pen mates on focal individual and have a genetic origin (Bijma, 2010). In general, in previous studies on IGEs, no indicators of social behavior were considered. In these studies, it was assumed an equal degree of interaction between each pair of mates in a group (Muir, 2005; Cantet and Cappa, 2008). Chen *et al*. (2009) and Cantet and Cappa (2008) reported limitations in these studies derived from collinearity both between direct and IGEs and between pen effects and IGEs. Some research has been performed to overcome this problem like Alemu *et al*. (2014 and 2016) who assumed that an individual expresses its indirect effect on each of its social partners depending on whether they are relative or not. They did not find important differences in interactions among related versus unrelated individuals. So, our aim with this study is to further contribute to alleviate this point.

Animals change their feeding behavior when they are housed in groups (Harb *et al*., 1985; Nielsen *et al*., 1995), or these changes can occur due to social interactions (Goetsch *et al*., 2010) or dominance ranking (Val-Laillet *et al*., 2008; Walker *et al*., 2008). Young (2012) concluded that feeding behavior may greatly affect animal growth and feed efficiency. Nowadays, feeding behavior traits of pigs housed in groups can be evaluated using data from electronic feeders. We hypothesize that correlations (or rank correlations in consecutive weeks) between feeding behavior phenotypes of pen mates can reflect the social structure and therefore part of the interactions between them. In the present study, we propose an alternative implementation of the AMs including IGEs, aiming at alleviate collinearity through the use of feeding behavior variables to approach a specific degree of interaction between each pair of animals sharing the same pen.

## Material and methods

### Animals and management

Animals used in this study come from a closed Duroc maternal line that was reproductively closed in 1991 (Tibau *et al*., 1999). Data used for this study consisted on the average daily gain (ADG) records obtained from 2008 until 2013 in a total of 663 animals, distributed over 57 pens and subjected to the same management at IRTA’s experimental facilities. Only records from 10- to 25-week-old animals, housed in pens with 10 to 14 pen mates were kept for analyses. The full pedigree file of the line included 5013 individuals produced from 883 sires and 2914 dams whereas the animals with phenotype observations were sired by 29 boards and they were offspring of 259 sows. Animals were fed *ad libitum* on a standard diet containing 15.9% CP, 4.5% fiber, 5.2% fat, 0.7% lysine and 0.2% methionine.

Individual feed intake, feeding time and a number of visits were recorded using IVOG^®^ feeding stations (Insentec, Markenesse, The Netherland) with single-space feeder for each pen. Feeding behavior variables were derived from the edited feeder data by computing variables by 1-h blocks during the day, resulting in the following feeding behavior variables: feeding rate (FR, average feed intake per unit of time, in g/min); feeding frequency (FF, total number of visits to the feeder per day, in units); occupation time (OT, time at feeder trough per day, in min/day) and time between consecutive visits (FInt, the mean of time between two consecutive visits per day, in min/day).

A preliminary descriptive study of feeding behavior variables was done by computing means, SD and phenotypic correlations between these variables. Moreover, the correlation between the ranks generated on the basis of these variables for each animal in consecutive periods of 2 weeks during the fattening period was computed to assess the stability of the ranks along the fattening period.

### Statistical models and data analyses

Three models were used to estimate genetic parameters for ADG in pigs during the fatting period:

(1) Animal model 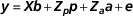
where ***y*** is the vector of ADG records; ***b*** the systematic effects vector including batch (six levels), initial age (covariate), final age (covariate) and number of pigs per pen (covariate); ***p*** the vector of random pen effects; ***a*** the vector of random additive genetic effects; ***X***, ***Z***_*p*_ and ***Z***_***a***_ are incidence matrices for systematic, pen and additive genetic effects, respectively, and ***e*** the vector of residuals. Random factors were assumed to be independent among them. Prior distribution of additive genetic values and pen effects were 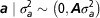 and 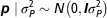, respectively, where ***A*** is the matrix of coefficients of relatedness between individuals, 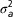 the additive genetic variance and 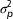 the pen effect variance. Flat priors were assumed for systematic effects (***b***) and variance components associated to random effects: 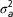, 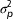 and 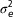.
(2) Animal model with classical indirect genetic effects 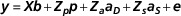
where ***y***, ***X***, ***b***, ***Z***_*p*_, ***p*** and ***e*** are defined as previously in AM; ***a***_***D***_ the vector of direct genetic effects (corresponding to the individual breeding value for the trait), with incidence matrix ***Z***_*a*_ relating observed data to the individual direct breeding value for the trait; and ***a***_***S***_ the vector of IGEs on the phenotype, being ***Z***_*s*_ the incidence matrix linking the observed data to the indirect breeding values of their group members. Animals sharing the same pen are assumed to interact equally between them (Muir, 2005; Cantet and Cappa, 2008). This way, elements of ***Z***_s_ are 1 for each pair of animals sharing the same pen and 0 otherwise, that is each individual interact with *n*-1 of its group members, where *n* is the size of the group. In the AM-IGE model, total breeding value (TBV) of individual *i* corresponds to TBV_*i*_=*a*_*Di*_+(*n*−1)*a*_*Si*_, and the variance of this component is 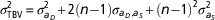. Under AM-IGE model the proportion of phenotypic variance explained by total breeding value variation (defined as 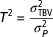) may exceed one, as according to Bijma *et al*. (2007) total phenotypic variation under this model is 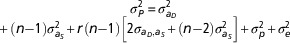, where *r* is the average relationship coefficient between all pairs of individuals within a pen which was equal to 0.13. The prior distribution of the additive genetic values in AM-IGE model was 
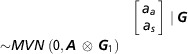
, where ***A*** is as in AM model, ⊗ denotes the Kronecker product, and ***G*** is the additive genetic (co)variance matrix: 
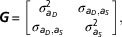
where 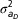 is the direct genetic variance, 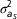 is the indirect genetic variance and 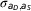 the covariance between direct and IGEs. Flat priors were assumed for these variance components: 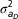, 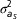 and 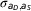. Prior distributions of the other random and systematic effects were as in AM model.
(3) Animal model fitting indirect genetic effects with specific degree of interaction between pair mates

The suggested model was equivalent to the AM-IGE model but assuming specific degree of interaction between each pair of animals. The specific levels of interaction were computed on the basis of the observed phenotypes for feeding behavior variables, considering the difference in feeding behavior variables between a pair of animals as indicators of the degree of competition between them. In this case the model was: 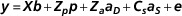
where the only different term with respect to IGE was the incidence matrix for the IGEs, here denoted as ***C***_***s***_. Elements of ***C***_***s***_ were defined as the specific degree of social interaction between each pair of animals sharing the pen, being 0 when the animals are in different pens. Elements of ***C***_***s***_ were computed as standardized Euclidean distance between each pair of individuals. Several alternatives for obtaining these Euclidean distances were tested depending upon the behavioral trait considered:

(a) When a unique feeding behavior variable was considered (models denoted as AM-IGE_FR_, AM-IGE_FF_, AM-IGE_OT_ and AM-IGE_FInt_) the Euclidean distance was 
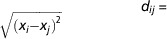
, where *x*_*i*_ and *x*_*j*_ are the standardized records of animals *i* and *j* for the feeding behavior variable, respectively. Elements of ***C***_***s***_ were computed as 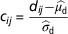, where 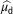 and 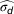 were mean and standard deviation across all mate pairs.
(b) When all standardized feeding behavior variables were considered jointly (model AM-IGE _ALL_), the Euclidean distance between animals was defined as:

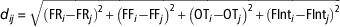
also, the standardized Euclidean distance was calculated as in the previous section as 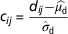. Under these models total genetic variance and phenotypic variances are also differed from AM-IGE model: total breeding values variation was 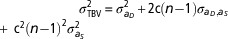, and the total phenotypic variation 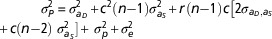, where *c* represents an average degree of social interaction between pen mates.

For all analyses, statistics of the marginal posterior distributions of all unknown parameters were obtained using the Gibbs Sampling algorithm. The software used for Gibbs Sampling was gibbs2f90 (Misztal *et al*., 2002). Chains of 3 000 000 samples were run and the first 300 000 iterations were discarded; one sample every 10 iterations was retained.

### Model comparison

#### Models were compared by means of four different criteria

(a) Accuracy of parameter estimation, measured as the standard deviation of the marginal posterior distributions for genetic parameters (Misztal and Wiggans, 1988; Van Raden and Wiggans, 1991).
(b) Deviance Information Criterion (DIC) that indicates the goodness of fit of the model to the observed data, penalizing by model complexity (Spiegelhalter *et al*., 2002).
(c) The predictive ability of each model estimated by 8-folds cross-validation. The total data set was partitioned into eight subsets leaving out one subset every fold (validation set). These validation sets were created randomly sampling one record per pen each time, thus the validation sets always included 57 animals, being different individuals in the different folds. Estimates and predictions obtained for fixed and random effects were used to predict observations of animals left out; then correlation coefficient between predicted and observed ADG was computed to assess the predictive ability of the models (Arlot and Celisse, 2010).
(d) Finally, the percentage of coincidence between top 10% of the population according to either EBV (in the AM model) or TBV (in the AM-IGE and AM-IGE_i_) models were computed. Correlations between the full genetic rankings between the compared models were also estimated.

## Results

### Feeding behavior variables as indicators of social interaction

Descriptive statistics and phenotypic correlations for feeding behavior variables and ADG are presented in Table 1. It was found low correlations between the considered feeding behavior traits and ADG. The phenotypic correlations between feeding behavior variables are an important aspect to be considered when analyzing feeding behavior variables. Any hypothetical behavior pattern of animals throughout the feeding behavior data could be detected exploring these correlations, by assessing a biological meaningful relationship between the variables. The estimated correlation coefficient between FR and FF was positive (0.56), that is the animals that visited many times the feeders tend to have greater feed intake per unit of time. Consequently, both FR and FF correlated negatively with FInt (−0.60 and −0.64, respectively). Moreover the negative correlation between FR and OT (−0.20), despite it is low, suggests that these animals with high feed rate, spend less time per day at the feeders. In summary, a classification on the basis of these variables would separate animals that eat calmly (low feed rate), in a reduced number of visits, with large interval between them, and occupying the feeder for a long time – assumed to be the dominants – from animals which eat fast, many times per day, having short interval between visits and spending less time in the feeder through the day assumed to be the subordinated. On the basis of these results, we hypothesize that distances based on these behavioral traits could somehow define the social structure within a pen.

**Table 1:** Descriptive statistics and phenotypic correlation coefficients of feeding behavior variables and average daily gain (*n*=663) in maternal Duroc pigs

						Correlation coefficients			
	Mean	Minimum	Maximum	SD	CV	FF	OT	FInt	ADG
FR (g/min)	54.45	21.3	104.65	14.69	27.14	0.56	−0.20	−0.60	0.21
FF (visits/day)	10.72	4.54	25.88	3.05	28.51		0.22	−0.64	0.11
OT (min/day)	63.34	36.56	104.63	10.49	16.45			−0.40	0.32
Time between visits (FInt, h)	4.05	1.90	9.88	1.22	26.19				0.01
ADG (g/day)	837.9	494.2	1069.96	96.42	–				

FF=feeding frequency; OT=occupation time; ADG=average daily gain.

In order to assess the capability of behavior variables to define a constant social structure across the fattening period, feeding behavior phenotypes were measured for 2-week periods and Spearman’s correlations between ranks of animals in consecutive periods were computed (Table 2). The estimated rank correlations varied from 0.67 to 0.86 and generally increased with age. If social rank within a group is defined on the basis of these behavioral traits, these moderate to high values would suggest that social structure within a pen is defined at early stages after mixing, and subsequently maintained relatively constant for the rest of the fattening period.

**Table 2:** Spearman’s rank correlation coefficients (*ρ*) between behavior variables in consecutive periods of 2 weeks (P_i_), from 10–24 weeks in maternal Duroc pigs

Behavior variables	*ρ*(P_1_,P_2_)	*ρ*(P_2_,P_3_)	*ρ*(P_3_,P_4_)	*ρ*(P_4_,P_5_)	*ρ*(P_5_,P_6_)	*ρ*(P_6_,P_7_)
Feeding rate (g/min)	0.67	0.78	0.80	0.84	0.82	0.80
Feeding frequency (visits/day)	0.71	0.80	0.83	0.86	0.82	0.85
Occupation time (min/day)	0.75	0.76	0.81	0.82	0.83	0.82
Time between visits (h)	0.72	0.80	0.83	0.85	0.80	0.85

### Genetic parameters

Posterior means for (co)variance components and genetic parameters of ADG with the implemented models are shown in Table 3. As expected, a medium to a large estimate of heritability for ADG was obtained with the single AM. Results from IGE model evidenced the existence of IGEs on ADG, which entails additional heritable variance. Despite its low estimated variance (0.004), the contribution of IGEs to total heritable variation is 1.16 times higher than the direct genetic effects and supposes up to 58% of the ADG phenotypic variance. Nonetheless, the estimated proportion of heritable variance (*T* ^2^) obtained with AM-IGE model was only slightly greater than the heritability estimated in AM. It should be taken into account that components involved in *T* ^2^ and in *h* ^2^ are different. In this particular case, negative correlation between direct and IGEs (−0.39) is the cause for the limited magnitude of *T* ^2^.

**Table 3:** Posterior mean (standard deviation) for (co)variance components and genetic parameters of average daily gain of maternal Duroc pigs obtained with the animal model (AM), the animal model with classical indirect genetic effects (AM-IGE) and five different indirect genetic effects animal models considering different feeding behavior traits to define the specific degree of social interaction between two individuals (AM-IGE_i_)

	AM	AM-IGE	AM-IGE_FR_	AM-IGE_FF_	AM-IGE_FInt_	AM-IGE_OT_	AM-IGE_ALL_
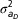	0.35 (0.13)	0.38 (0.11)	0.36 (0.11)	0.40 (0.12)	0.38 (0.12)	0.37 (0.12)	0.37 (0.11)
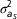	–	0.004 (0.003)	0.002 (0.002)	0.001 (0.001)	0.003 (0.002)	0.005 (0.002)	0.004 (0.002)
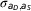	–	−0.018 (0.02)	0.009 (0.009)	−0.005 (0.008)	0.005 (0.009)	−0.017 (0.009)	−0.025 (0.012)
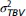	–	0.43 (0.25)	0.36 (0.15)	0.40 (0.12)	0.38 (0.13)	0.37 (0.13)	0.38 (0.11)
*h* ^2^or*T* ^2^	0.47 (0.15)	0.54 (0.29)	0.51 (0.14)	0.55 (0.13)	0.53 (0.14)	0.53 (0.14)	0.53 (0.12)
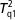	–	–	0.51 (0.18)	0.75 (0.20)	0.65(0.20)	1.29 (0.25)	1.24 (0.30)
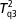	–	–	0.72 (0.22)	0.53 (0.19)	0.68 (0.22)	0.53 (0.21)	0.33 (0.19)
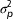	0.04 (0.02)	0.04 (0.03)	0.03 (0.02)	0.04 (0.02)	0.04 (0.02)	0.04 (0.02)	0.05 (0.03)
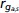	–	−0.39 (0.47)	0.312 (0.28)	−0.33 (0.48)	0.113 (0.31)	−0.41 (0.21)	−0.65 (0.24)

AM-IGE_FR_, AM-IGE_FF_, AM-IGE_OT_, AM-IGE_FInt_ and AM-IGE_ALL_=animal models including indirect genetic effects using feeding rate, frequency, time, interval and overall index to define the specific interaction degree between two individuals; 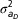
=direct genetic variance; 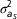
=indirect genetic variance; 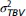
=total heritable variance;
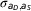
=covariance between direct and indirect genetic effects; *h* ^2^and *T* ^2^ proportion of total heritable variance in the model; 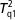, 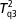
=*T* ^2^ at the first and third quartile of the interaction degree scale; 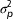
=pen environmental variance; 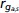
=genetic correlation between direct and indirect genetic effects.

An important point to check is estimating *T* ^*2*^ at a different degree of social interaction. *T* ^2^ evaluated at the mean of the interaction degree (mean of standardized Euclidean distance), that is zero, obtained with the five AM-IGE_*i*_ models resulted similar among them, and also similar to that reported by the classical AM-IGE model. However, a deeper analysis of its components and the posterior distribution for this parameter shows important differences between models. To show the differences between *T* ^2^ estimates at extreme interaction degrees under the different AM-IGE_*i*_ models; *T* ^2^ were estimated at the first (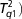 – low interaction degree – and third (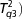 quartile – large interaction degree – of the distribution of the elements of C matrix (Table 3). As C is a centered variable, zero (the mean) represents the average interaction degree. It was observed, in some cases, important differences between these two estimates of *T* ^*2*^. This was the case of the models where the degree of interaction was defined by OT and the global behavior index while no important differences between the two estimates of *T* ^2^ were observed for the other AM-IGE_i_ models (Table 3). The differences between estimates of T^2^ at first and third quartiles were 0.76 (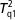
=1.29 minus 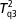
=0.53) in the case of AM-IGEOT and 0.91 (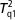
=1.24 minus 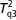
=0.33) in the case of AM-IGEALL, whereas in the other model’s differences were much lower. This supposes that for models AM-IGE_OT_ and AM-IGE_ALL,_ the changes in the ratio between total heritable and phenotypic variations, as function of the degree of social interaction between the animals, are more important than for the others. These changes can be traced back to the magnitude of the genetic parameters; for example, for the aforementioned two models the highest and more negative genetic correlations between indirect and direct genetic effects were observed and also for these two models the highest indirect genetic variances were estimated.

A key point in the AM-IGE models, both considering or not a variable degree of interaction between mates, is the genetic correlation between direct and IGEs. Estimates for this parameter differed in sign and magnitude between the six AM-IGE models, depending on the definition of the interactions. When the joint index of all behavioral variables was used to define the interaction between individuals (AM-IGE_ALL_) negative correlation between indirect and direct genetic effects was observed. However, in general, our results do not allow us being conclusive about the sign of this correlation given large standard errors for the covariance.

Finally, it is worthy to emphasize the reduction in the estimation errors (posterior SD) observed when the no-constant social interaction between animals was considered (AM-IGE_*i*_ models) when compared with the standard AM-IGE model.

### Models comparison

The quality of fit of the analyzed models was compared by means of DIC (Table 4). In general, the lowest values of DIC clearly point out the superiority of any model fitting IGEs over the standard AM for fitting ADG data. When the six different social models are compared, DIC values point out a better fit of models with non-constant social interaction degree between pen mates (AM-IGE_i_ models *v.* AM-IGE model) apart from the AM-IGE_FR_ model. Among AM-IGE_i_ models, the best fit was obtained when the social interaction degree was defined on the basis of daily time (AM-IGE_OT_) in the feeder.

**Table 4:** Model comparison by deviance information criteria (DIC) and the correlation between observed and predicted average daily gain of maternal Duroc pigs records (*r*_y,ŷ_) from an 8-fold cross-validation test, mean (SD) across folds

	AM	AM-IGE	AM-IGE_FR_	AM-IGE_FF_	AM-IGE_FInt_	AM-IGE_OT_	AM-IGE_ALL_
DIC	1402.07	1348.69	1376.08	1338.59	1330.77	1304.74	1321.54
*r*_y,ŷ_	0.52 (0.12)	0.53 (0.16)	0.55 (0.09)	0.52 (0.07)	0.54 (0.05)	0.56 (0.05)	0.58 (0.04)

AM=animal model; AM-IGE=animal model including classical indirect genetic effects; AM-IGE_FR_, AM-IGE_FF_, AM-IGE_OT_, AM-IGE_FInt_ and AM-IGE_ALL_=animal model including indirect genetic effects but using feeding rate, frequency, time, interval and overall index to define the specific degree of interaction between two individuals.

Table 4 also shows the correlation between observed and predicted ADG records obtained by cross-validation. Despite correlations in Table 4 reveal small differences between the predictive ability of these models, AM-IGE_i_ models were again superior to both AM and standard AM-IGE model but for AM-IGE_FF_. Among AM-IGE_i_ models, when the social interaction was defined as the Euclidean distance for OT, we obtained the best prediction ability. The model AM-IGE_OT_ showed correlations between observed and predicted phenotypes 5.7% and 7.6% superior to the AM-IGE and AM models, respectively.

Differences between models regarding parameter estimates, the goodness of fit and predictive ability are expected to have consequences in the genetic ranking generated by these models. Table 5 shows the percentage of coincidence in genetic rankings between top 10% animals and between the full rankings. Important differences (rank correlation lower than 0.8) between genetic rankings are inferred from these results for AM-IGE_OT_ and AM-IGE_ALL_, and the rest of the models, Surprisingly, TBVs rankings from AM-IGE_FR_, AM-IGE_FF_ and AM-IGE_FInt_ models were more coincident with EBVs ranking from AM than with TBV from standard AM-IGE model. The highest differences between rankings were observed when comparing the AM-IGE_OT_ with the rest of the models (<44% of matches in top 10% animals). In a lesser extent, also the genetic ranking derived from AM-IGE_ALL_ was far from the other models (<65% of matches in top 10% animals). It is worth mentioning that both AM-IGE_OT_ and AM-IGE_ALL_ models showed the highest accuracy of genetic parameters estimates, the lowest DIC and the best predictive ability.

**Table 5:** Percentage of coincidence between top 10% animals (above diagonal) and rank correlations between genetic rankings (below diagonal) in maternal Duroc pigs

	EBV_animal_	TBV_CONST_	TBV_FR_	TBV_FF_	TBV_FInt_	TBV_OT_	TBV_ALL_
EBV_animal_	–	0.70	0.79	0.89	0.76	0.44	0.52
TBV_CONST_	0.89	–	0.68	0.68	0.58	0.41	0.52
TBV_FR_	0.94	0.85	–	0.83	0.79	0.39	0.59
TBV_FF_	0.98	0.86	0.95	–	0.83	0.41	0.62
TBV_FInt_	0.91	0.80	0.92	0.93	–	0.44	0.65
TBV_OT_	0.61	0.59	0.61	0.61	0.63	–	0.48
TBV_ALL_	0.69	0.69	0.79	0.79	0.82	0.71	–

EBV_animal_=estimated breeding value using Animal model; TBV_CONST_=Total breeding value using Animal model including classical indirect genetic effects; TBV_FR_, TBV_FF_, TBV_OT_, TBV_Fint_ and TBV_ALL_=Total breeding value using Animal model including indirect genetic effects but using feeding rate, frequency, time, interval and overall index to define the specific degree of interaction between two individuals, respectively.

Finally, correlations between direct, indirect and total breeding values were estimated considering predictions for all animals with phenotypic observation (Table 6). These results aim to explore the possible consequences of selecting for each component of the total breeding value. The correlation between direct and indirect breeding values was negative in AM-IGE, AM-IGE_FF_, AM-IGE_OT_ and AM-IGE_ALL_ (−0.85, −0.38, −0.63 and −0.89, respectively), whereas positive correlations were observed for AM-IGE_FR_ and AM-IGE_FInt_. These estimates agree with the genetic correlations reported in Table 4.

**Table 6:** Correlation (cor) between direct genetic (DBV), social genetic (SBV), and total breeding values (TBV) for average daily gain (g) of maternal Duroc pigs considering predictions for all the animals with a phenotypic observation

	AM-IGE	AM-IGE _FR_	AM-IGE _FF_	AM-IGE _FInt_	AM-IGE _OT_	AM-IGE _ALL_
cor (DBV,SBV)	−0.85	0.69	−0.38	0.35	−0.63	−0.89
cor (DBV,TBV)	0.86	0.97	0.97	0.94	0.67	0.68
cor (TBV,SBV)	−0.46	0.84	−0.18	0.64	0.13	−0.30

AM-IGE=Animal model including classical indirect genetic effects; AM-IGE_FR_, AM-IGE_FF_, AM-IGE_OT_, AM-IGE_FInt_ and AM-IGE_ALL_=animal model including indirect genetic effects but using feeding rate, frequency, time, interval and overall index to define the specific degree of interaction between two individuals.

The obtained correlations between direct genetic effect and total breeding values were high in all models, ranging from 0.67 to 0.97. Conversely, the correlation between IGEs and total breeding values varied notably across models. The highest values for this correlation were observed in AM-IGE_FR_ (0.84) and AM-IGE_FInt_ (0.64), where as in AM-IGE, AM-IGE_FF_ and AM-IGE_ALL_ this correlation was moderate and negative (−0.46, −0.18 and −0.30, respectively).

## Discussion

In the present study, ADG records from Duroc pigs fattened in groups were fitted to several AMs which differed in the way the interaction degree between pen mates was computed. Results obtained from such models and comparison to AM model confirmed the existence of IGEs for growth when pigs are housed in groups. These results are in line with previous studies reporting evidences of indirect effects on growth rate not only in pigs (Arango *et al*., 2005; Chen *et al*., 2008), but also in other species such as quail (Muir *et al*., 2013), shrimp (Luan *et al*., 2015), rabbit (Piles *et al*., 2017) and Nile tilapia (Khaw *et al*., 2016).

All the previous results demonstrated the importance of including IGEs in models for analysis of ADG, as ignoring these effects might cause biased estimates and suboptimal responses to selection; therefore it could lead to an increase in the frequency of dominant or aggressive individuals in the population.

Despite the reported evidence of genetic variance due to IGEs, no relevant differences regarding the proportion of phenotypic variance due to genetic effects were observed between AM-IGE and AM models, with *T* ^2^ estimates ranging from 0.51 to 0.55 whereas heritability estimated with AM was 0.47. Conversely, in previous studies on pig growth, the proportion of variance due to additive genetic effects increased substantially when IGEs were included in the model. Bergsma *et al*. (2008) obtained heritability estimates ranging from 0.25 to 0.36 with a classical AM, whereas using a AM-IGE the proportion of variance due to total breeding values increased to 0.71, whereas Bergsma *et al*. (2013) reported substantially lower *T* ^*2*^ value (0.34) for ADG but the *T* ^*2*^ still ~50% greater than suggested by classical heritability. In line with these result, Chen *et al*. (2009) reported estimates of heritability for ADG ranging from 0.13 to 0.34, whereas *T* ^*2*^ ranged from 0.38 to 2.34. Similar results were reported by Wilson *et al*. (2009) in deer mice: when IGEs for rearing rate and reciprocal latency rate were considered, total heritable variation increased from 0.10 to 0.60 and from 0.05 to 0.56, respectively. Unlike these studies, our low value of *T* ^*2*^ estimates relies on the negative covariance between direct and indirect effects (Table 3), which is constraining part of the additional variance captured by IGEs. The existence of a negative genetic correlation between direct and indirect effects could have detrimental consequences on selection for ADG. If IGEs are not considered in the genetic evaluation, selection procedure could conduct to an increasing of the aggressiveness and competition between animals, yielding a reduced or even negative genetic response to selection (Griffing, 1967; Bijma *et al*., 2007). In previous studies, Bergsma *et al*. (2008) obtained a positive but low correlation between indirect and direct genetic effects (0.20), whereas Chen *et al*. (2009) observed that such correlation for ADG in pigs varied substantially across populations (from −0.37 to 0.74), and generally had large standard errors. Moreover, in domestic chickens, Peeters *et al*. (2012) reported moderate to the high negative correlation between indirect and direct genetic effects in crossbreds (−0.37 and −0.83), being low and not significantly different from zero this correlation in purebred animals (0.20 and −0.28).

The standard implementation of AM-IGE represents an alternative to fit indirect effects in genetic evaluations but without any further consideration about the actual degree of interaction between group mates. These models have been proved to be useful in different selection experiments for pecking behavior in laying hens (Kjaer *et al*., 2001), BW in Japanese quail (Muir, 2005) or IGEs on ADG in pigs (Camerlink *et al*., 2014). Camerlink *et al*. (2014) did not find any relevant effect on growth after one generation of divergent selection for IGE of growth, and in the contrary to what would be expected high IGE pigs showed higher stomach wall damage score. In the contrary to this pattern, Camerlink *et al*. (2015) found that high IGE pigs showed less biting behavior than animals with lower IGE for growth.

However, there still exists some controversy over its use due to the collinearity between direct and IGEs and between pen and indirect effects (Cantet and Cappa, 2008; Chen *et al*., 2009). These statistical limitations to adequately disentangle random effects in AM-IGE models entail large errors in genetic parameters estimations and breeding value predictions, which might penalize the genetic progress of any selection process. In our data set, in addition to these issues, we have the problem of its limited size, thus in order to evaluate the role that the prior information might have played in the final estimates we performed an EM-REML estimation of the parameter in model AM-IGE to assess whether these new estimates match the Bayesian estimates reported in Table 3. Considering the large errors of our estimates (Table 3), it can be concluded that the match between both approaches is reasonable as the uncertainty region of the Bayesian estimates (Table 3) always included the EM-REML estimates, for example, the EM-REML estimate of the correlation between direct and IGE was −0.30, whereas that from the Bayesian approach was −0.39. In addition to this, a simulation study was done to assess whether the estimated parameters could be satisfactorily recovered in the case they would be used for generating the data. We observed that the confidence intervals created around the mean of the EM-REML estimates across 10 replicates +/− 2 times the standard deviation across the replicates always included the true values used in the simulation for all the variance components. With these two pieces of information, it seems clear that although our estimates have large errors, they do not seem to be subject to artifacts associated either to the prior information used in the Bayesian analysis or to a lack of convergence during the estimation processes.

One of the hypotheses of this study is that defining specific interactions between each pair mates based on some variable could alleviate the collinearity between effects, thus improving the accuracy of parameter estimation and breeding value prediction. The plausibility of this hypothesis was illustrated in the experimental designs presented by Cantet and Cappa (2008) in which animals are moved from their pens, and differential degrees of interaction between pair mates were defined according to time they have been sharing the pen. Under this theoretical design, these authors showed an improvement in the estimation efficiency, with lower errors in the variance components estimates. Also, Alemu *et al*. (2014, 2016) attempted to improve AM-IGE models considering the degree of social interaction to be depended on whether a pair of partners are family or not, or whether they are from the same sex or not. Their implementation in mink did not report relevant differences between social interaction effects in related *v.* unrelated minks, but they were observed when the interaction degree was defined on sex basis. These studies also showed that this new implementation of the social AM was statistically superior to the classical AM-IGE in terms of goodness of fit of the data.

In the light of these works, in the present study, we aimed at proposing an alternative implementation to the AM-IGE, the AM-IGE_i_ models, defining specific degrees of social interaction between pair mates on the basis of feeding behavior variables. An important advantage of using AM-IGE_i_ models is that these models would allow assessing for the consequences of different degrees of interaction among pen mates on *T* ^2^ estimates and subsequently on the expected response to selection. A direct statistical result of fitting AM-IGE_i_ models is an important reduction in the estimation errors of the genetic parameters. For example, the marginal posterior SD of the genetic correlation between indirect and direct genetic effects decreased from 0.47 with AM-IGE to 0.21 with AM-IGE_OT_. As differences in variance components involved in a correlation influence the magnitude of estimation errors (Falconer and Mackay, 1996), it is worth mentioning that in both AM-IGE and AM-IGE_OT_ models estimates of (co)variances for indirect and direct genetic effects were similar. Other criteria used for model comparison in the present study, DIC and predictive ability, equally point to superiority of models fitting a non-constant degree of social interaction defined by feeding behavior phenotypes.

Among the five AM-IGE_i_ proposed models, AM-IGE_OT_, that is that defining the degree of interactions between pen mates on the basis of the OT, is the best one, followed by the model considering an index with all the feeding behavior variables (AM-IGE_ALL_). In fact, AM-IGE_ALL_ was the only one providing estimates of genetic correlation between direct and indirect effects that can be said to be statistically different from zero.

Our descriptive study of five feeding behavior traits clearly points out that ranking the animals accordingly to these variables approaches a within-pen hierarchy that remains relatively constant across time and can be assumed to define a certain social structure in our population. Previously, several behavioral studies assessing a degree of antagonistic interactions between animals (i.e. observing who wins in fights) had reported associations between feeding behavior and actual position in social hierarchy in pigs (Nielsen *et al*., 1995), dairy cows (Val-Laillet *et al*., 2008) and goat (Gipson *et al*., 2006; Jørgensen *et al*., 2007). In the same line, Nielsen *et al*. (1995) showed that growing pigs modify their feeding behavior when social interactions change, for example FR increase and feeder occupation decreased when the level of competition increases by introducing more animals in the pen. In dairy cows, Harb *et al*. (1985) showed that animals fed in groups modify their FR with respect to those fed individually; particularly the submissive cows that increase their FR. Results presented here seem to endorse the suitability of using feeding behavior variables to define the differential degree of social interaction between pen mates.

We can thus conclude that considering feeding behavior variables to define differential interaction degree across pairs of mates helps to improve the performance of models fitting IGEs for genetic evaluation.

## Conclusions

With regard to ADG in our Duroc population, the classical AM in which an individual’s phenotype is assumed to be controlled by direct genetic effects alone have the potential to give misleading expectations for selection as IGEs seems to play an important role in the definition of the trait. Using specific levels of competition or interaction for each pair of animals, defined by the feeding behavior variables of the pair, reduce the SE of the estimated genetic parameters and improved the predictability of the model. Genetic ranks based on total breeding value predictions greatly vary among considered models, so different selection decisions are expected to be taken. As a final remark, it can be indicated that the models in which IGEs were fitted as a function of the OT or as a function of an index including all feeding behavior variables are preferred to any other model for genetic evaluation of growth in our Duroc line.
